# An Orthogonal Protection Strategy for the Synthesis of Conotoxins Containing Three Disulfide Bonds

**DOI:** 10.3390/md23040168

**Published:** 2025-04-14

**Authors:** Hengyu Zhang, Lai Yue Chan, Huanhuan Zhang, Tao Jiang, David J. Craik, Wenqing Cai, Rilei Yu

**Affiliations:** 1 Key Laboratory of Marine Drugs, Chinese Ministry of Education, School of Medicine and Pharmacy, Ocean University of China, Qingdao 266003, China; zhy2645@stu.ouc.edu.cn (H.Z.); zhanghuanhuan@stu.ouc.edu.cn (H.Z.); jiangtao@ouc.edu.cn (T.J.); 2 Laboratory for Marine Drugs and Bioproducts, Qingdao National Laboratory for Marine Science and Technology, Qingdao 266237, China; 3 Institute for Molecular Bioscience, Australian Research Council Centre of Excellence for Innovations in Peptide and Protein Science, The University of Queensland, Brisbane, QLD 4072, Australia; angeline.chan@imb.uq.edu.au (L.Y.C.); d.craik@imb.uq.edu.au (D.J.C.); 4 Shandong Academy of Pharmaceutical Sciences, Jinan 250100, China

**Keywords:** orthogonal oxidation method, three disulfide bonds, conotoxins

## Abstract

Disulfide bonds are crucial for stabilizing bioactive peptides such as conotoxins. We have developed a method for synthesizing conotoxins with three disulfide bonds using Mob, Trt, and Acm protection groups for regionally selective synthesis. This approach enabled the efficient synthesis of peptides with the desired disulfide bond connectivities independent of their sequences. Using our strategy, we synthesized five conotoxins, achieving yields of 20–30%. The results demonstrate the potential of our method for synthesizing complex peptides with multiple disulfide bonds.

## 1. Introduction

Conotoxins are small, disulfide-bond-rich neurotoxic peptides with diverse structures, potent biological activity, and high selectivity [1]. They specifically bind to a wide range of ion channels, transporters, receptors, and other molecular targets, making them promising leads for the development of drugs to treat neurological diseases [2,3,4]. The ω-conotoxin MVIIA (ziconotide), which contains three disulfide bonds, was the first conotoxin approved for the treatment of acute and chronic pain [5].

Disulfide bonds are crucial for the stability and activity of conotoxins [6,7]. They impose conformational constraints, enhancing the stability of these peptides and improving their metabolic and thermal resistance [8]. As disulfide-rich peptides, conotoxins typically exhibit high selectivity and affinity for their molecular targets. However, their high cysteine content complicates their chemical synthesis. Although strategies for synthesizing conotoxins with two disulfide bonds have been developed [4,9], the synthesis of conotoxins with three or more disulfide bonds remains challenging. Achieving isoform specificity in disulfide bond formation during chemical synthesis is particularly challenging for such peptides, with syntheses typically resulting in low oxidative folding yields.

**Figure 1:**
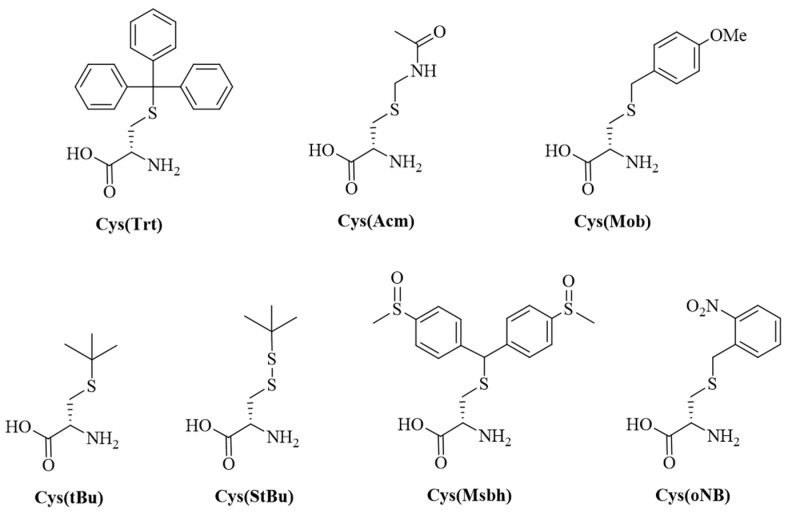
Structures of the thiol protection groups.

Non-selective disulfide bond formation in REDOX buffers can be a viable approach. Typically, peptides are kept at low concentrations, and mild oxidants such as oxidized glutathione/glutathione (GSSG/GSH) are employed to mimic the intracellular disulfide bond formation environment [10], resulting in thermodynamically stable products. However, this method does not ensure the correct formation of disulfide bonds in the final product. By contrast, the orthogonal synthesis approach achieves directed folding by employing the orthogonal protection of pairs of cysteine residues and selectively removing protective groups under different reaction conditions. Various cysteine-protecting groups have been developed for the orthogonal synthesis of peptides containing three disulfide bonds (Figure 1). Acid-labile protective groups, such as trityl (Trt), are widely used alongside acetamidomethyl (Acm), which is unstable in the presence of iodine. Tert-Butyl (tBu) serves as a classic sulfhydryl protecting group, stable under TFA and REDOX conditions, and can be effectively removed using PhS(O)Ph/CH_3_SiCl_3_ in TFA [11]. 4,4′-Bis (dimethylsulfinyl) benzhydryl (Msbh) is highly stable under acidic and REDOX conditions and is often removed by NH_4_I/DMS/TFA [12]. However, both groups are usually incompatible with tryptophan, limiting their applicability. The reducing-agent-labile tert-butylsulphenyl (StBu) has several advantages, including reduced dimerization and broad orthogonality. However, it is often difficult, and sometimes impossible, to remove using reducing agents [13]. Often, deprotection on the resin before TFA treatment is necessary, leading to by-products and low yields. A light-sensitive cysteine protection strategy using 1,2-nitrobenzyl (oNB) is frequently employed but may encounter side reactions and sequence dependency during synthesis [14,15]. Overall, synthesizing peptides with three disulfide bonds using orthogonal protection remains challenging.

**Figure 2:**
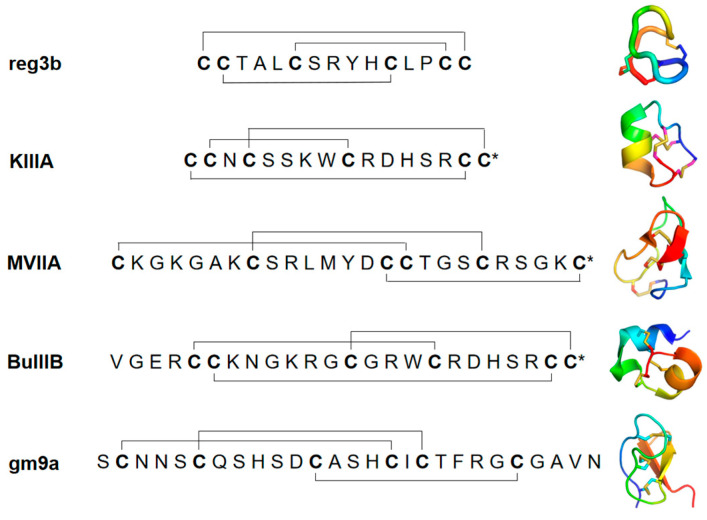
Primary structures and 3D NMR structures of five exemplar conotoxins containing three disulfide bonds.

4-Methoxybenzyl (Mob) has long been used as a thiol protection group and is commonly employed in Boc-SPPS. It is unstable in strong acids and can be removed by HF or boiling TFA [16]. However, its use is limited by these harsh removal conditions. In 2012, a method for S-Mob removal was developed using TFA/thioanisole/DTNP [17]. However, the reaction products are required to be treated with a thiol, which can disrupt existing disulfide bonds. Additionally, the inclusion of DTNP results in enhanced reactivity towards other protecting groups, such as Acm and tBu groups, complicating its application in orthogonal protection synthesis. More recently, increasing the temperature and extending the reaction time in the presence of TIS has also proven effective for S-Mob removal [18]. In 2019, a single Mob protection group was employed to protect one cysteine residue in insulin chain A [19]. However, the application of a single Mob group has limitations in synthesizing a single peptide chain. To overcome the synthesis challenges of disulfide-rich peptides and develop a universal orthogonal oxidation strategy, in this study, we utilized two Mob protection groups for the orthogonal synthesis of single-chain peptides containing three disulfide bonds under mild TFA conditions, in conjunction with Trt and Acm groups, for the first time. The introduction of two Mob groups presents the challenge of optimizing the deprotection conditions for both; however, the method developed in this study significantly improves upon previous approaches by reducing sequence dependency and successfully synthesizing five conotoxins with three disulfide bonds (Figure 2). The symbol * in Figure 2 indicates C-terminal amidation.

## 2. Results and Discussion

### 2.1. S-Mob Deprotection Conditions

**Figure 3:**
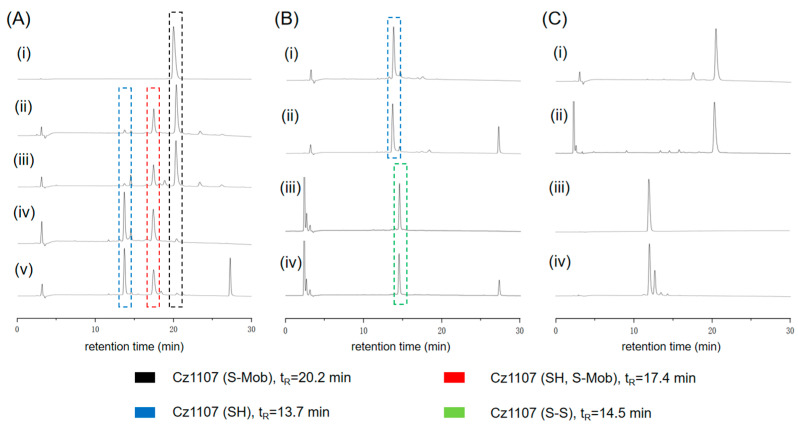
Exploration of S-Mob deprotection using conotoxin Cz1107 as a template. (**A**) Linear Cz1107 with two Mob groups was incubated under different conditions for 1 h at 40 °C: (**i**) untreated; (**ii**) TFA; (**iii**) TFA/H_2_O (95:5); (**iv**) TFA/TIS/H_2_O (95:2.5:2.5); (**v**) TFA/thioanisole/H_2_O (95:2.5:2.5). (**B**) Linear Cz1107 with Mob groups was incubated for 3 h at 40 °C with TIS or thioanisole added: (**i**) TFA/TIS/H_2_O (95:2.5:2.5); (**ii**) TFA/thioanisole/H_2_O (95:2.5:2.5); (**iii**) iodine added to the product of (**i**); (**iv**) iodine added to the product of (**ii**). (**C**) Orthogonality exploration of S-Mob, S-Acm, and S-Trt. (**i**) Treated Cz1107(S-Mob) with reagent K for 2 h at room temperature; (**ii**) treated Cz1107(S-Mob) with iodine at room temperature for 1 h; (**iii**) linear Cz1107 with two Acm groups; (**iv**) treated Cz1107(S-Acm) with TFA/TIS/H_2_O (95:2.5:2.5) for 1 h at 40 °C.

In order to explore the removal conditions of S-Mob and its compatibility with S-Trt and S-Acm in the orthogonal oxidation strategy, it is necessary to verify the reaction conditions using a template peptide. We used Cz1107, a conotoxin containing just a single disulfide bond, to explore the favorable S-Mob deprotection conditions, with HPLC employed as an analytical tool (Figure 3; Table 1). Thiols were protected with Mob and incubated in various TFA solutions. After 1 h at 40 °C, the linear peptide was converted into the product Cz1107(SH) with exposed thiol groups and the intermediate Cz1107(SH, S-Mob) generated by the partial removal of the S-Mob group (Figure 3A). S-Mob was unstable in pure TFA, yielding 4% product and 25% intermediate, and in 95% TFA/H_2_O, yielding 8% product and 20% intermediate. The reaction rate significantly increased with TIS (48% product, 36% intermediate) or thioanisole (45% product, 33% intermediate). After 3 h at 40 °C, S-Mob groups were fully removed with TIS or thioanisole, resulting in trace disulfide-bonded product (Figure 3(Bi–Bii)). The addition of iodine facilitated disulfide bond formation, achieving high yields of 86% with TIS (Figure 3(Biii)) and 82% with thioanisole (Figure 3(Biv)). The results of Cz1107 (S-Mob) deprotection under different conditions are summarized in Table 1. The MS characterization of Cz1107 is provided in the Supplementary Materials (Figure S1). All yields in this study were HPLC yields.

These results indicated that S-Mob can be completely removed using TFA in the presence of TIS or thioanisole. In contrast, S-Trt is sensitive to TFA (1–10%) as an acid-labile protecting group [20,21], which is eliminated usually during resin cleavage. S-Mob, however, is relatively stable during this process. Notably, S-Mob can also be partially removed under conventional cleavage conditions and, thus, it should be avoided for excessive temperature and high concentrations of TFA during cleavage. Here, we adopt reagent K [22] for the reaction at room temperature (Figure 3(Ci); 12%). Given the small amount of removal and the significant difference in hydrophobicity between the by-products and the desired product, there is a negligible impact on the overall yield.

Acm as an orthogonal pair of sulfhydryl protective groups is a suitable option and can be removed with iodine. Linear peptide with Mob groups remained stable in the presence of iodine (Figure 3(Cii)). In contrast, S-Acm was unstable in TFA solution at 40 °C (Figure 3(Civ), 37% deprotection), showing increased removal over time. The by-product that removes the S-Acm groups could be difficult to separate from the desired product due to their similar physicochemical properties. The partial lability of S-Acm under S-Mob deprotection conditions necessitates its removal prior to S-Mob deprotection.

### 2.2. Synthesis of Conotoxin reg3b

**Scheme 1:**
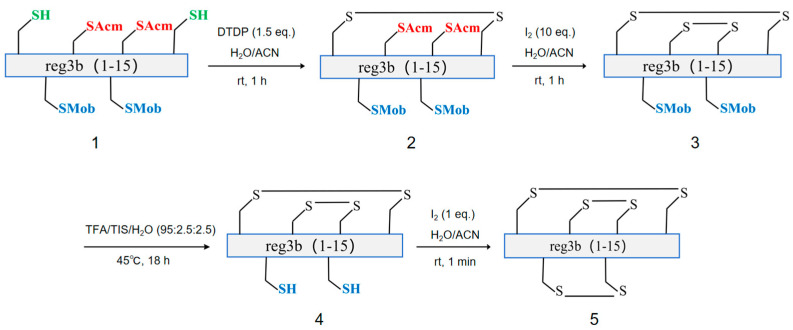
Synthesis of conotoxin reg3b.

**Figure 4:**
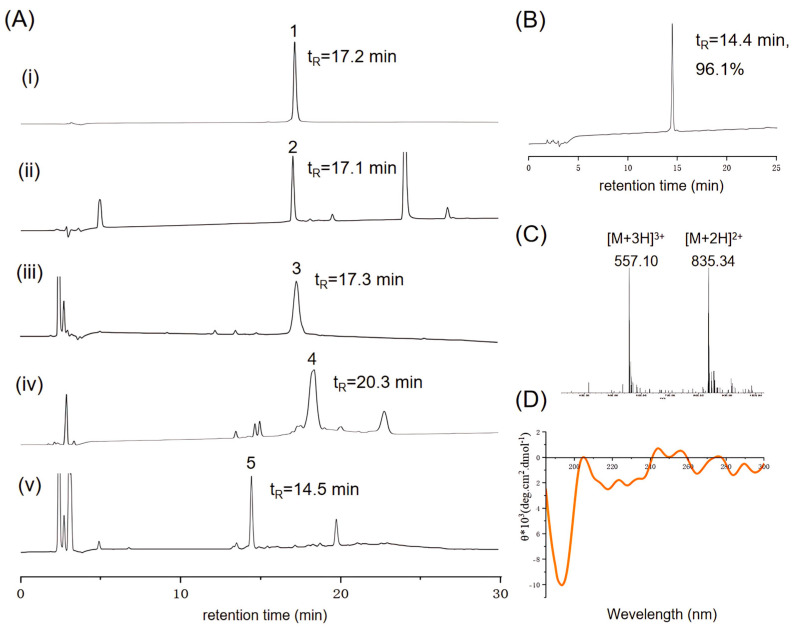
Synthesis of conotoxin reg3b. (**A**) HPLC chromatograms of the reaction progression: (**i**) linear peptide with one pair of free thiols and two pairs protected by S-Mob and S-Acm groups, respectively; (**ii**) DTDP was added to facilitate the formation of the first disulfide bond; (**iii**) the second disulfide bond was formed through I_2_-mediated S-Acm oxidation; (**iv**) treated with TFA/TIS/H_2_O (95:2.5:2.5) at 45 °C for 18 h to remove the S-Mob groups, and the product was not purified; (**v**) a small amount of iodine was added to the reaction products in (**iv**). (**B**) HPLC chromatogram of synthetic reg3b. (**C**) MS characterization of synthetic reg3b. (**D**) CD spectrum of synthetic reg3b. The HPLC elution gradient from 10% ACN/H_2_O to 70% ACN/H_2_O took 30 min for samples (**i**–**iii**) and 60 min for samples (**iv**,**v**).

Following the trial with Cz1107, we next developed a synthetic scheme for the three-disulfide conotoxin reg3b (Scheme 1). The process began with the addition of 4,4′-dipyridyl disulfide (DTDP) to form the first disulfide bond, achieving a 71.4% yield (Figure 4(Aii)). Excess iodine was then used to remove the S-Acm groups, yielding a second disulfide bond with 90.2% (Figure 4(Aiii)). The intermediate containing two disulfide bonds was incubated in TFA/TIS/H_2_O (95:2.5:2.5) at 45 °C. The structural rigidity from disulfide bond formation decreased the S-Mob removal rate, reaching a maximum yield of 59% after 18 h, and the product can be used in the next reaction without purification (Figure 4(Aiv)). HPLC was used to monitor the reaction progress, showing that TIS was more effective for longer peptides than thioanisole, which produced more by-products. Finally, iodine oxidized the last pair of thiols, producing the target peptide with three disulfide bonds (Figure 4 (Av), 46.8% yield).

This strategy yielded reg3b with 96% purity (Figure 4B) and an overall yield of 30%. The synthetic reg3b was confirmed by ESI-MS, and the CD spectrum indicated random coil characteristics as reported in the NMR studies (Figure 4C,D) [23]. Additional mass spectra data are provided in the Supplementary Materials (Figure S2).

### 2.3. Synthesis of Conotoxin MVIIA

**Scheme 2:**
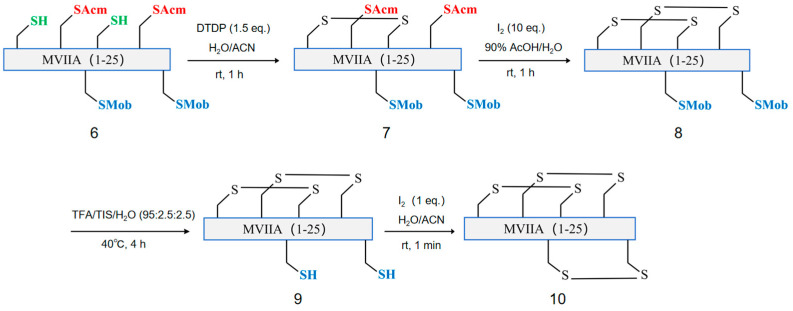
Synthesis of conotoxin MVIIA.

Among the known conotoxin sequences, those with three disulfide bonds constitute the largest proportion, predominantly featuring cysteine frameworks III (as exemplified by reg3b) and VI/VII [9]. The conotoxins within framework VI/VII, characterized by the stable inhibitory cysteine knot (ICK) motif, include ziconotide (MVIIA), which is currently employed as an analgesic medication. MVIIA exhibits a distinct backbone and disulfide bonding pattern compared to reg3b. To verify the generality of our synthetic method, we chose to synthesize MVIIA following the successful synthesis of reg3b (Scheme 2).

**Figure 5:**
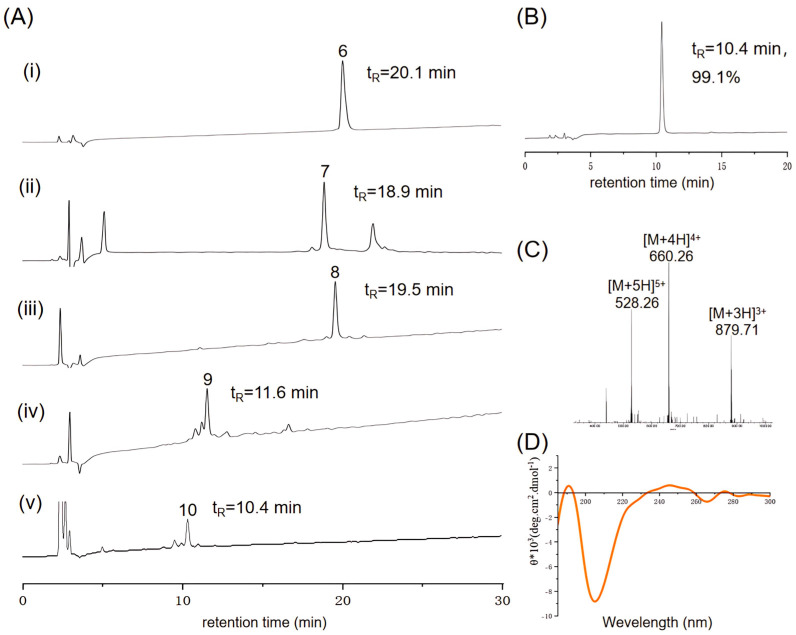
Synthesis of conotoxin MVIIA. (**A**) HPLC chromatograms of the reaction progression: (**i**) linear peptide with one pair of free thiols and two pairs protected by S-Mob and S-Acm groups; (**ii**) DTDP was added to facilitate formation of the first disulfide bond; (**iii**) the second disulfide bond was formed through I_2_-mediated S-Acm oxidation; (**iv**) treated with TFA/TIS/H_2_O (95:2.5:2.5) at 40 °C for 4 h to remove the S-Mob groups, and the product was not purified; (**v**) a small amount of iodine was added to the reaction products in (**iv**). (**B**) HPLC chromatogram of synthetic MVIIA. (**C**) MS characterization of synthetic reg3b. (**D**) CD spectrum of synthetic MVIIA.

The synthesis of MVIIA also employed DTDP to facilitate the first disulfide bond formation (Figure 5(Aii), 60.1% yield). Iodine was then utilized to mediate the formation of the second disulfide bond (Figure 5(Aiii), 78.1% yield). Unlike other synthetic tricyclic peptides, this solvent system for iodine oxidation utilized a 90% AcOH/H_2_O mixture to enhance the yield, in contrast to the H_2_O/ACN system previously employed. During the oxidation of the third pair of thiols, the yield of S-Mob de-protection peaked at 51% after treatment with TFA/TIS/H_2_O (95:2.5:2.5) at 40 °C for 4 h (Figure 5(Aiv)). After the addition of iodine, the third disulfide bond was successfully formed with a yield of 44.7% (Figure 5(Av)). Overall MVIIA was obtained with 99% purity (Figure 5B) and a yield of 21.0%. The CD spectrum of synthetic MVIIA exhibited a weak positive peak at 192 nm and a distinct negative peak at 205 nm, suggesting a typical β-sheet structure (Figure 5D), as reported in the NMR studies and the previous CD results [24,25,26]. Additional mass spectra data can be found in the Supplementary Materials (Figure S3).

### 2.4. Comparisons of Secondary H^α^ Chemical Shifts

**Figure 6:**
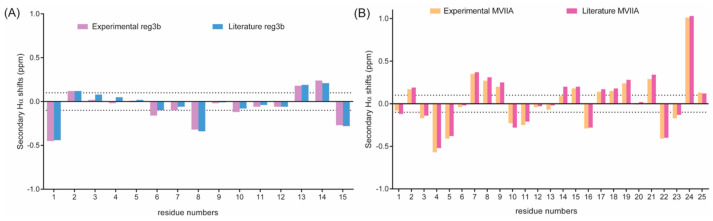
Secondary H^α^ chemical shifts of reg3b and MVIIA. (**A**) Comparison of the secondary H^α^ chemical shifts of experimental reg3b with literature values obtained from Franco et al., 2018 [23] (**B**) Comparison of the secondary H^α^ chemical shifts of experimental MVIIA with literature values obtained from Basus et al., 1995 [27]. Three or more consecutive residues with secondary chemical shifts greater than +0.1 ppm or less than –0.1 ppm (dotted lines) indicate potential structured regions within the peptide.

A detailed two-dimensional NMR analysis by NMR showed that the chemical shifts of reg3b and MVIIA in this work were identical within experimental error to the chemical shifts reported in the literature for these compounds [23,27], thus demonstrating that the molecules were correctly folded with the correct disulfide connectivity (Figure 6).

### 2.5. Application to the Other Three Conotoxins

We also applied the aforementioned synthesis strategy to three additional conotoxins with distinct structures and disulfide bond connection patterns. Following treatment with DTDP, iodine, a mixture of TFA/TIS/H_2_O (95:2.5:2.5), and iodine again, the final product was obtained with a purity exceeding 95%. The total yields were 26.1%, 23.4%, and 26.5%, respectively, with detailed synthetic schemes and results available in the Supplementary Materials (Figures S4–S12 and Figure S14). Notably, the rigid structures enhanced the resistance of S-Mob to TFA. During the synthesis of tricyclic peptides, the S-Mob removal became more efficient as the sequence length increased. MVIIA, BuIIIB, and gm9a, containing 25, 24, and 27 amino acid residues, respectively, achieved maximum yields after 4 h of reaction at 40 °C. In contrast, KIIIA and reg3b required 18 h at 45 °C to reach maximum yields. Furthermore, the reaction rate was positively correlated with temperature. For instance, KIIIA required over 24 h to reach its maximum yield at 40 °C, but this time decreased to 18 h at 45 °C and 12 h at 50 °C, with the structure remaining stable at all test temperatures (Figure S13).

## 3. Materials and Methods

### 3.1. General Information

All amino acids and resins were purchased from GL Biochem Ltd. (Shanghai, China), except for Fmoc-Cys(Mob)-OH (CAS 141892-41-3), which was obtained from Bidepharm (Shanghai, China). Preparative RP-HPLC was conducted using FL162040-H100-A0021 system, while analytical RP-HPLC was carried out on EasySep 1010 system. The peptide preparation utilized a 5C18-MS-I column (20 × 250 mm) with detection at 214 nm, whereas a 5C18-MS-II column (4.6 ID × 250 mm) was employed for analytical RP-HPLC. The HPLC solvents were prepared as follows: Solvent A was 0.05% TFA in H_2_O/ACN (*v*/*v*: 90:10), and solvent B was 0.05% TFA in ACN/H_2_O (*v*/*v*: 90:10). The flow rate for preparative RP-HPLC varied from 6 to 10 mL/min using a linear gradient from 10% to 50% Solvent B over 60 min. The analytical RP-HPLC was conducted with a linear gradient of 0.5–2% per minute, progressing from 10% to 70% Solvent B at a flow rate of 1 mL/min.

### 3.2. Synthesis of Linear Peptides

Linear peptides were synthesized using standard Fmoc solid-phase peptide synthesis (SPPS) protocols. Coupling reactions were performed with Fmoc-protected amino acids (4 eq.), HCTU (4 eq.), and DIEA (8 eq.) in DMF for 40 min, followed by deprotection of the Fmoc group with 20% piperidine in DMF for 20 min. After each reaction, the resin was thoroughly washed with DMF (2 × 10 mL), DCM (2 × 10 mL), and DMF (2 × 10 mL). The progress of SPPS reaction was monitored using the Kaiser test. Peptide synthesis utilized Rink-amide MBHA resin (0.42 mmol/g) and 2Cl-resin pre-loaded with the first amino acid (0.35 mmol/g), following previously described methods [28,29]. In the final step, the resin was washed with DCM (6 × 10 mL), and the crude peptide was cleaved from the resin while simultaneously deprotecting the side chains except for the S-Acm and S-Mob protection groups by treating it with a mixture of TFA/thioanisole/H_2_O/phenol/EDT (82.5:5:5:5:2.5) for 2 h at 25 °C. The peptide was then precipitated with cold diethyl ether twice and subsequently dissolved in H_2_O/ACN for preparative RP-HPLC purification.

### 3.3. Oxidative Folding of Peptides

All folding processes were conducted at a concentration of 2 mg/mL. DTDP was dissolved in a small amount of methanol and slowly added dropwise to the linear peptide in H_2_O/ACN under neutral conditions to form the first disulfide bond. The second disulfide bond was formed through I_2_-mediated S-Acm oxidation under acidic conditions. MVIIA was reacted in 90% acetic acid aqueous solution, while all other peptides were processed within the H_2_O/ACN system. After the reaction, ascorbic acid was added to quench the process, and the oxidized products were isolated via HPLC and lyophilized. The resulting oxidized peptides, containing two disulfides, were dissolved in TFA that also contained 2.5% *v*/*v* TIS and 2.5% *v*/*v* H_2_O. These solutions were kept in a shaker at 40 °C or 45 °C. After the reaction, the peptides were precipitated using cold diethyl ether and dissolved in H_2_O/ACN. A small amount of iodine was added to facilitate the formation of a third disulfide bond. Finally, the target product was purified by HPLC, and all reaction processes were monitored by MS.

### 3.4. Circular Dichroism (CD)

CD spectra were recorded at room temperature under a nitrogen atmosphere using a Jasco J-810 spectropolarimeter (JASCO, Tokyo, Japan). Measurements were taken over a wavelength range of 300–185 nm with a 1.0 mm path length cell, a bandwidth of 1.0 nm, and a response time of 2 s. Each spectrum values were averaged over three scans. Peptides were dissolved in H_2_O at a concentration of 0.2 mg/mL. The spectra are expressed as molar ellipticity ([θ]), calculated as follows: [θ] = 1000·mdeg/(l·c), where mdeg represents the raw CD data, c indicates the peptide’s molar concentration (mM), and l is the cell path length (mm).

### 3.5. NMR Spectroscopy

MVIIA and reg3b were analyzed in this study. Each peptide (~1 mg) was dissolved in 90% H_2_O/10% D_2_O, and 1 µL of a 1 mg/mL solution of 4,4-dimethyl-4-silapentane-1-sulfonic acid (DSS) was added as an internal reference. One-dimensional ^1^H spectra and two-dimensional spectra (TOCSY and NOESY) were acquired. All spectra were recorded on a Bruker Avance 600 MHz spectrometer at 298 K and were processed using TOPSIN 3.6.1 (Bruker) program. NMR data were analyzed using CCPNMR spectra assignment program (version 2.4.2) and all sequential assignments were performed according to the procedure described by Wishart et al. [30].

## 4. Conclusions

In summary, we developed a facile route for synthesizing conotoxins with three disulfide bonds through regionally selective synthesis. Peptides with three pairs of thiols protected by Trt, Acm, and Mob efficiently folded into desired disulfide connectivities in a sequence-independent manner, using conditions similar to conventional cleavage methods. This strategy enabled the synthesis of five conotoxins with different skeletons and three disulfide bonds, yielding target products of 20–30%. However, some shorter peptides required a longer reaction time, indicating future areas for improvement in deprotection conditions. Overall, this approach demonstrates versatility and simplicity, showcasing significant potential for the orthogonal protection synthesis of complex peptides with multiple disulfide bonds.

## Figures and Tables

**Table 1:** Recovery yield of Cz1107(S-Mob) for different conditions.

Entry	Experimental Conditions	Recovery Yield (%) ^a^
1	TFA, 1 h at 40 °C	71
2	TFA/H_2_O (95:5 *v*:*v*), 1 h at 40 °C	72
3	TFA/TIS/H_2_O (95:2.5:2.5 *v*:*v*:*v*), 1 h at 40 °C	16
4	TFA/thioanisole/H_2_O (95:2.5:2.5 *v*:*v*:*v*), 1 h at 40 °C	22
5	TFA/TIS/H_2_O (95:2.5:2.5 *v*:*v*:*v*), 3 h at 40 °C	3
6	TFA/thioanisole/H_2_O (95:2.5:2.5 *v*:*v*:*v*), 3 h at 40 °C	4
7	Reagent K, 2 h at RT	88
8	Iodine, 1 h at RT	97

^a^ HPLC proportion of Cz1107 (S-Mob) residual amount.

## Data Availability

The data underlying this study are available in the published article and its Supplementary Materials.

## Supplementary Materials

The following supporting information can be downloaded at: https://www.mdpi.com/article/10.3390/md23040168/s1, Table S1: MS characterization of synthetic peptides; Figures S1–S6 are the MS characterization diagrams of Cz1107, reg3b, MVIIA, KIIIA, gm9a, and BuIIIB, respectively; Figures S7–S9 are the synthetic schemes of KIIIA, BuIIIB, and gm9a, respectively; Figures S10–S12 are the HPLC diagrams of the synthesis processes of conotoxins KIIIA, gm9a, and BuIIIB, respectively; Figure S13: HPLC analysis of KIIIA reactions for S-Mob removal at different temperatures; Figure S14: HPLC traces of crude peptides after treatment with reagent K; Figure S15: 1H NMR of MVIIA and reg3b. References [23,31,32,33,34,35] are cited in the Supplementary Materials.

## Abbreviations

SPPS	Solid-phase peptide synthesis
HCTU	5-Chloro-1-[bis(dimethylamino)methylene]-1H-benzotriazolium 3-oxide hexafluorophosphate
DIEA	N,N-Diisopropylethylamine
DTDP	4,4′-Dipyridyl disulfide
Trt	Trityl
Acm	Acetamidomethyl
Mob	4-Methoxybenzyl
