# Supplementary material for: An Orthogonal Protection Strategy for the Synthesis of Conotoxins Containing Three Disulfide Bonds
Source: Mar Drugs. 2025 Apr 14;23(4):168. doi: 10.3390/md23040168 (PMC12028366; doi:10.3390/md23040168)
Supplement: Supplementary file 1 [file marinedrugs-23-00168-s001.zip › marinedrugs-3571937-supplementary.pdf]

Supplementary Materials

An Facile Orthogonal Protection Strategy for the Synthesis of Conotoxins Containing Three Disulfide Bonds

Hengyu Zhang,[1,2] Lai Yue Chan,[3] Huanhuan Zhang,[1,2] Tao Jiang,[1,2] David J. Craik,[3] Wenqing Cai [4,*] and Rilei Yu[1,2,*]

[1] Key Laboratory of Marine Drugs, Chinese Ministry of Education, School of Medicine and Pharmacy, Ocean University of China, Qingdao 266003, China; zhy2645@stu.ouc.edu.cn (H.Z.);
zhanghuanhuan@stu.ouc.edu.cn (H.Z.); jiangtao@ouc.edu.cn (T.J.)
[2] Laboratory for Marine Drugs and Bioproducts of Qingdao National Laboratory for Marine Science and Technology, Qingdao 266237, China
[3] Institute for Molecular Bioscience, Australian Research Council Centre of Excellence for Innovations in Peptide and Protein Science, The University of Queensland, Brisbane, QLD 4072, Australia; angeline.chan@imb.uq.edu.au (L.Y.C.); d.craik@imb.uq.edu.au (D.J.C.)
[4] Shandong Academy of Pharmaceutical Sciences, Jinan 250100, China
* Correspondence: caiwenqing@sdaps.cn (W.C.); ryu@ouc.edu.cn (R.Y.)

Table of Contents

1. MS Information and Spectra ······ S1-S6

2. Synthetic schemes of KIIIA, BuIIIB and gm9a ······ S7

3. The HPLC Chromatograms and CD Spectra ······ S8-S11

4. The HPLC of Crude Peptides ······ S12

5. 1H NMR of MVIIA and reg3b ······ S13

6. References ······ S14

1. MS Information and Spectra

All the following mass spectra data were collected using ESI-MS (Waters Acquity QDa).

Table S1. MS characterization of synthetic peptides.

Peptide #	Peptide Name	Exact Mass (Theoretical)	Mass (Observed)
1	Cz1107[31]	1106.48	1106.09
2	reg3b[23]	1668.58	1668.49
3	MVIIA[32]	2637.10	2636.39
4	KIIIA[33]	1882.64	1882.44
5	BuIIIB[34]	2761.17	2760.91
6	gm9a[35]	2797.00	2797.15

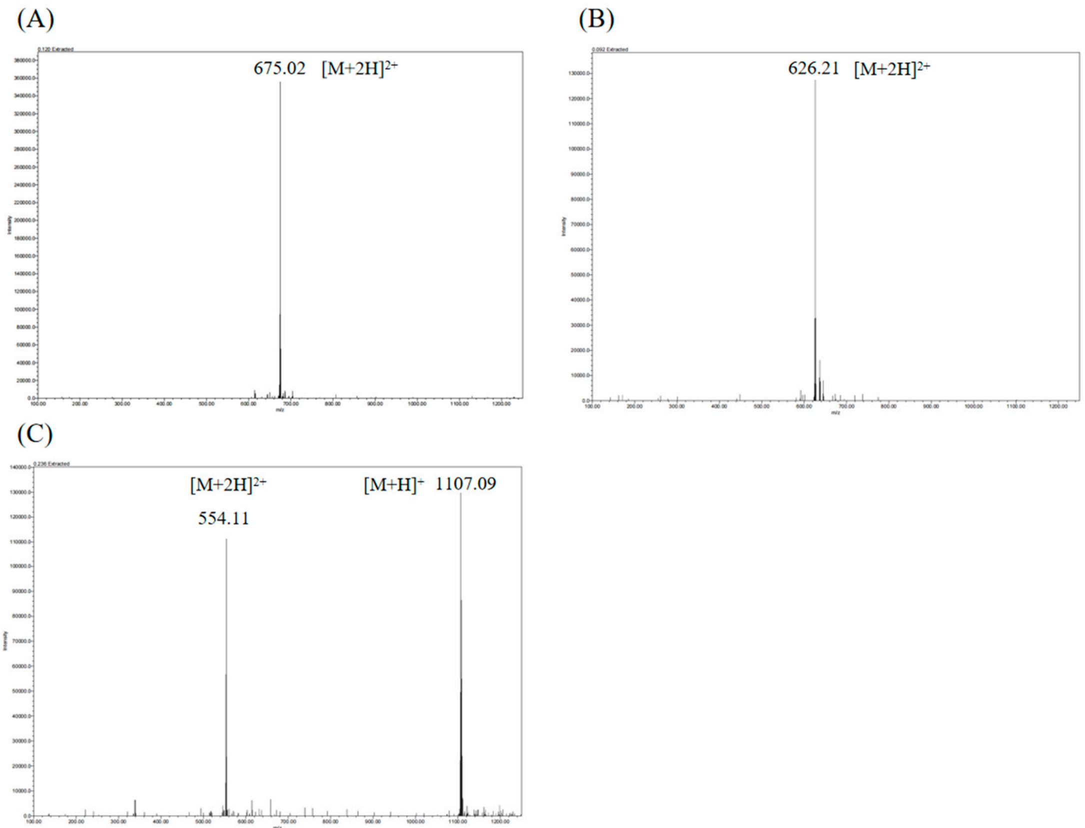

Figure S1. MS characterization of Cz1107. (**A**) The linear peptide with S-Mob groups; m/z 675.02 $[M + 2H]^{2+}$. (**B**) The linear peptide with S-Acm groups; m/z 626.21 $[M + 2H]^{2+}$. (**C**) Cz1107; m/z 1107.09 $[M + H]^{+}$, m/z 554.11 $[M + 2H]^{2+}$.

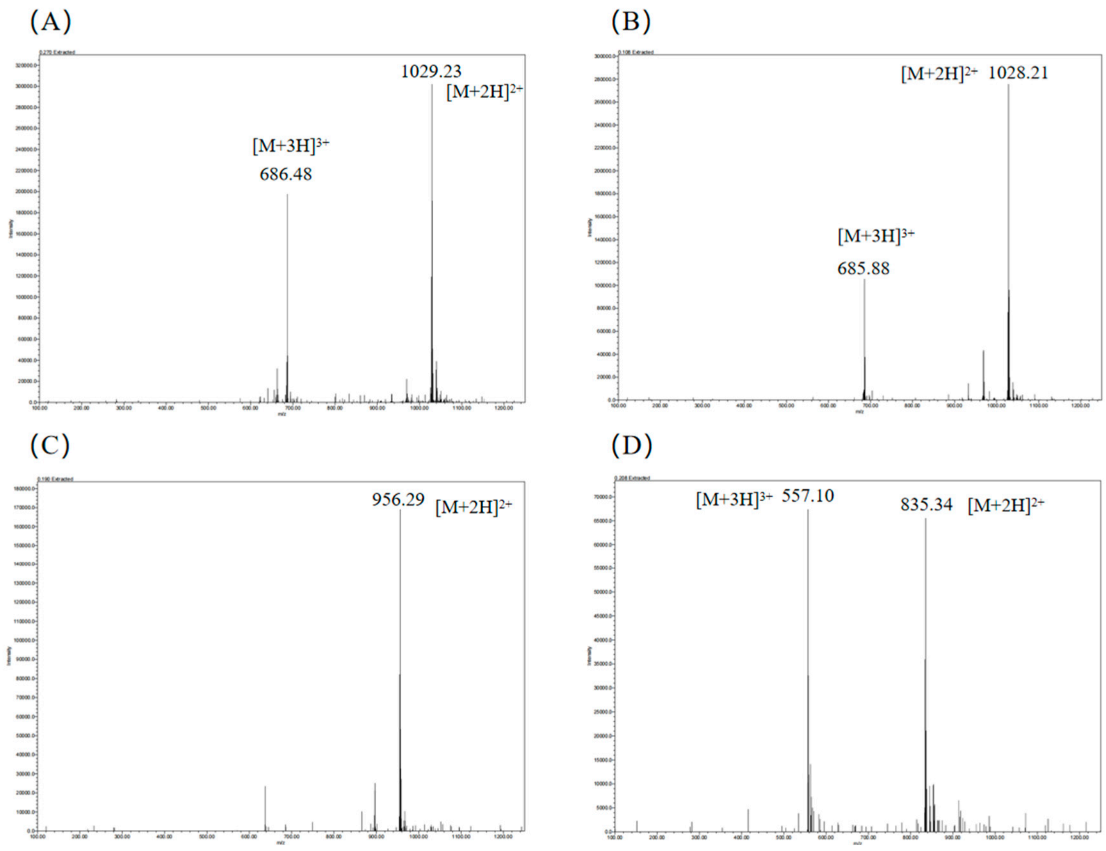

Figure S2. MS characterization of reg3b and its intermediates. (**A**) Linear peptide with one pair of free thiols and two pairs protected by S-Mob and S-Acm groups; m/z 1029.23 [M + 2H]$^{2+}$, 686.48 [M + 3H]$^{3+}$. (**B**) Monodisulfide intermediate after DTDP treatment; m/z 1028.21 [M + 2H]$^{2+}$, 685.88 [M + 3H]$^{3+}$. (**C**) Intermediate with two disulfide bonds after iodine treatment; m/z 956.29 [M + 2H]$^{2+}$. (**D**) reg3b; m/z 835.34 [M+2H]$^{2+}$, 557.10 [M + 3H]$^{3+}$. All reported m/z values correspond to fragmentation peaks.

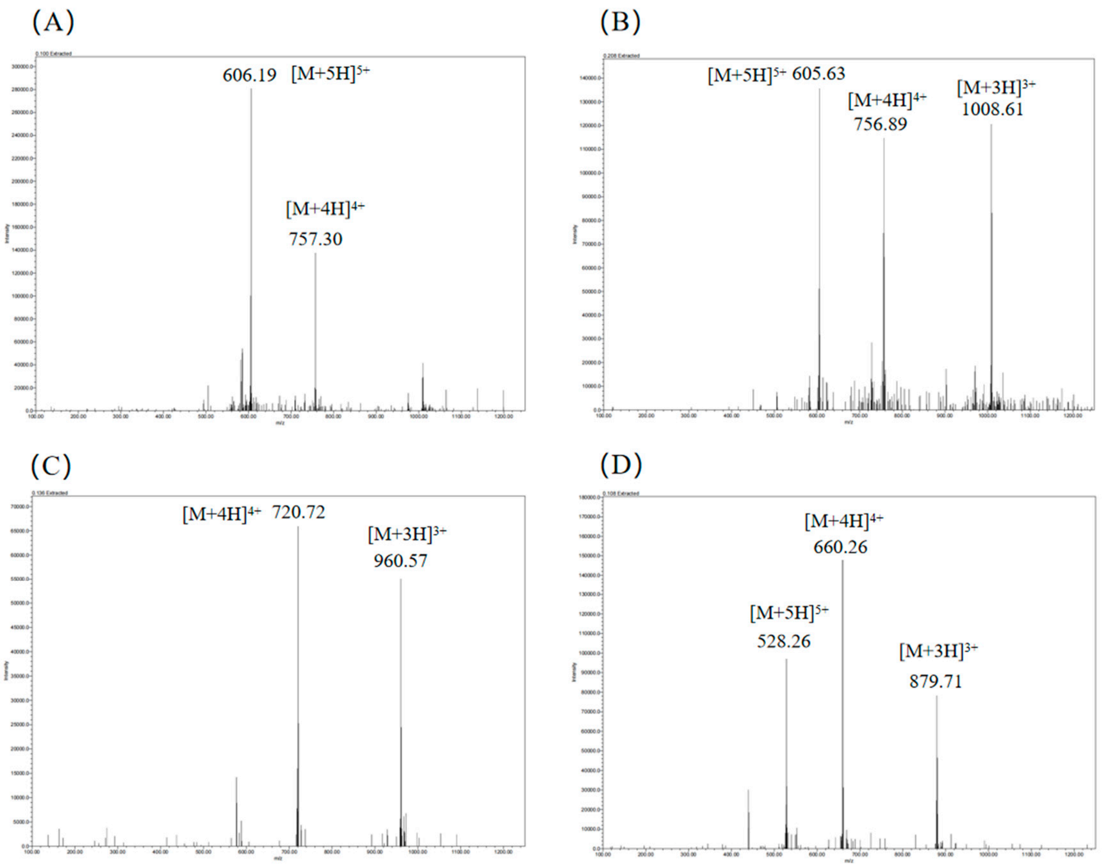

Figure S3. MS characterization of MVIIA and its intermediates. (**A**) Linear peptide with one pair of free thiols and two pairs protected by S-Mob and S-Acm groups; m/z 757.30 $[M + 4H]^{4+}$, 606.19 $[M + 5H]^{5+}$. (**B**) Monodisulfide intermediate after DTDP treatment; m/z 1008.61 $[M + 3H]^{3+}$, 756.89 $[M + 4H]^{4+}$, 605.63 $[M + 5H]^{5+}$. (**C**) Intermediate with two disulfide bonds after iodine treatment; m/z 960.57 $[M + 3H]^{3+}$, 720.72 $[M + 4H]^{4+}$. (**D**) MVIIA; m/z 879.71 $[M + 3H]^{3+}$, 659.44 $[M + 4H]^{4+}$, 528.26 $[M + 5H]^{5+}$. All reported m/z values correspond to fragmentation peaks.

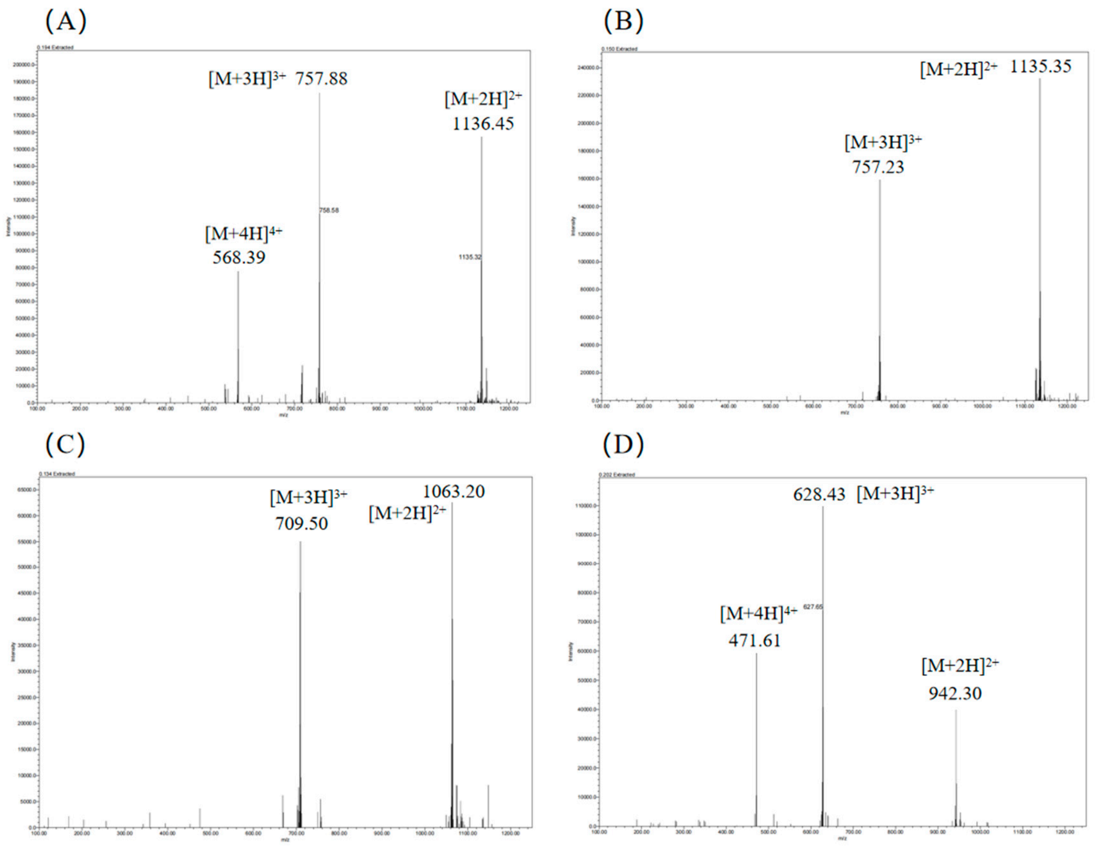

Figure S4. MS characterization of KIIIA and its intermediates. (**A**) Linear peptide with one pair of free thiols and two pairs protected by S-Mob and S-Acm groups; m/z 1136.45 $[M + 2H]^{2+}$, 757.88 $[M + 3H]^{3+}$, 568.39 $[M + 4H]^{4+}$. (**B**) Monodisulfide intermediate after DTDP treatment; m/z 1135.35 $[M + 2H]^{2+}$, 757.23 $[M + 3H]^{3+}$. (**C**) Intermediate with two disulfide bonds after iodine treatment; m/z 1063.20 $[M + 2H]^{2+}$, 709.50 $[M + 3H]^{3+}$. (**D**) KIIIA; m/z 942.30 $[M + 2H]^{2+}$, 628.43 $[M + 3H]^{3+}$, 471.61 $[M + 4H]^{4+}$. All reported m/z values correspond to fragmentation peaks.

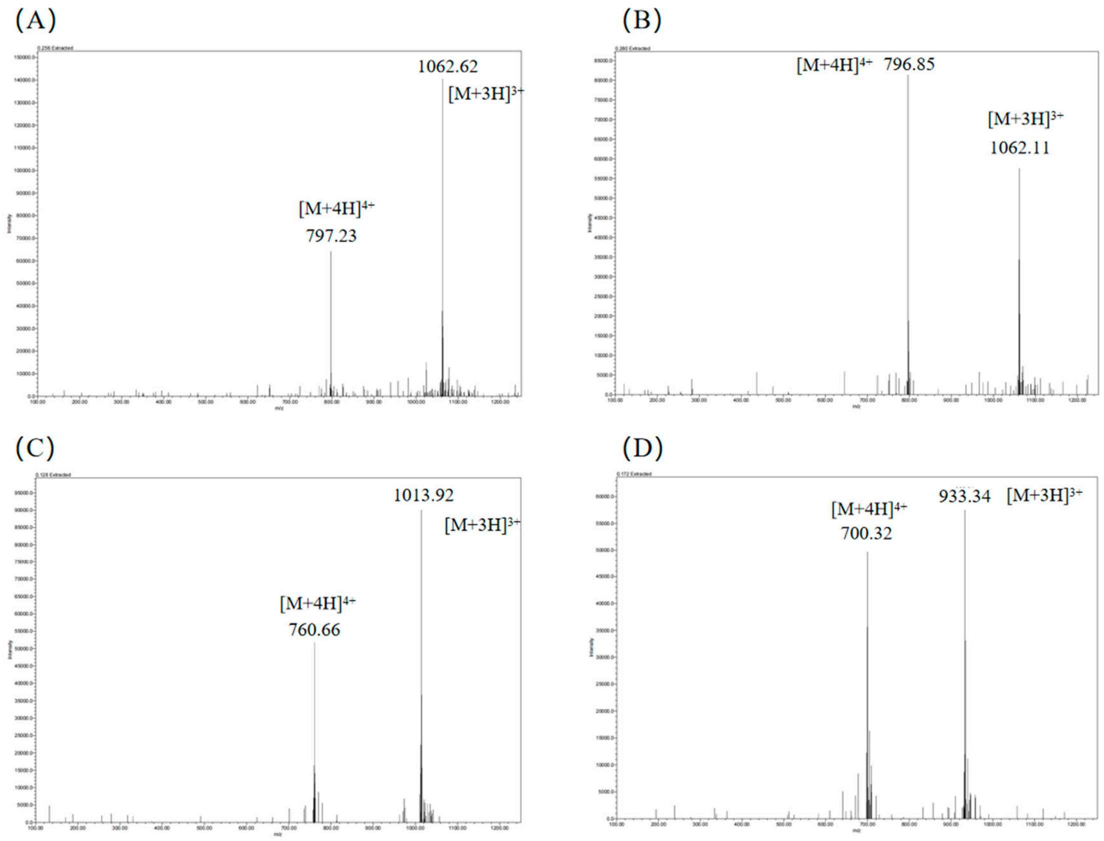

Figure S5. MS characterization of gm9a and its intermediates. (**A**) Linear peptide with one pair of free thiols and two pairs protected by S-Mob and S-Acm groups; m/z 1062.62 $[M + 3H]^{3+}$, 797.23 $[M + 4H]^{4+}$. (**B**) Monodisulfide intermediate after DTDP treatment; m/z 1062.11 $[M + 3H]^{3+}$, 796.85 $[M + 4H]^{4+}$. (**C**) Intermediate with two disulfide bonds after iodine treatment; m/z 1013.92 $[M + 3H]^{3+}$, 760.66 $[M + 4H]^{4+}$. (**D**) gm9a; m/z 933.34 $[M + 3H]^{3+}$, 700.32 $[M + 4H]^{4+}$. All reported m/z values correspond to fragmentation peaks.

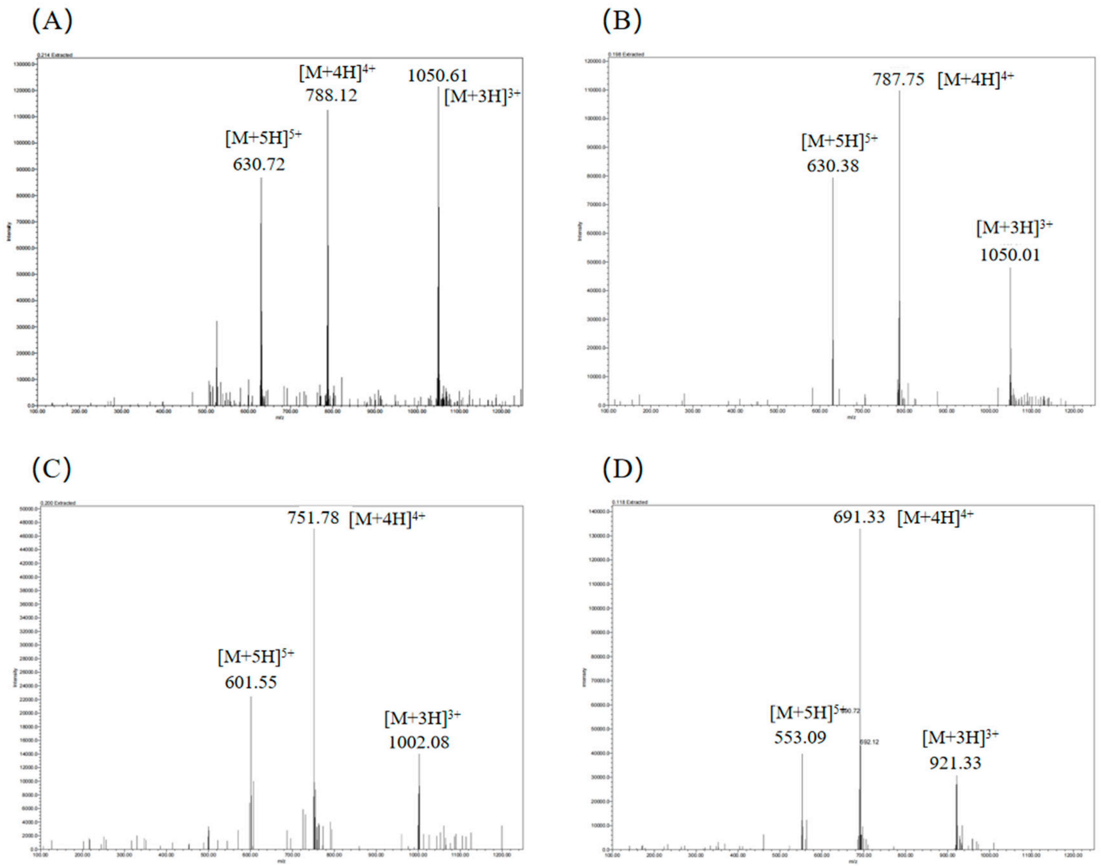

Figure S6. MS characterization of BuIIIB and its intermediates. (**A**) Linear peptide with one pair of free thiols and two pairs protected by S-Mob and S-Acm groups; m/z 1050.61 [M + 3H]$^{3+}$, 788.12 [M + 4H]$^{4+}$, 630.72 [M + 5H]$^{5+}$. (**B**) Monodisulfide intermediate after DTDP treatment; m/z 1050.01 [M + 3H]$^{3+}$, 787.75 [M + 4H]$^{4+}$, 630.38 [M + 5H]$^{5+}$. (**C**) Intermediate with two disulfide bonds after iodine treatment; m/z 1002.08 [M + 3H]$^{3+}$, 751.78 [M + 4H]$^{4+}$, 601.55 [M + 5H]$^{5+}$. (**D**) BuIIIB; m/z 921.33 [M + 3H]$^{3+}$, 691.33 [M + 4H]$^{4+}$, 553.09 [M + 5H]$^{5+}$. All reported m/z values correspond to fragmentation peaks.

2. Synthetic schemes of KIIIA, BuIIIB and gm9a

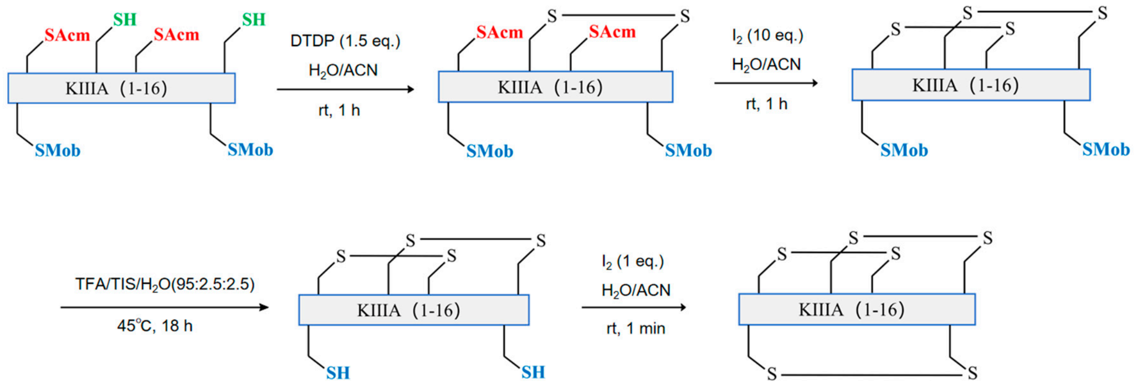

Figure S7. Synthetic scheme of conotoxin KIIIA.

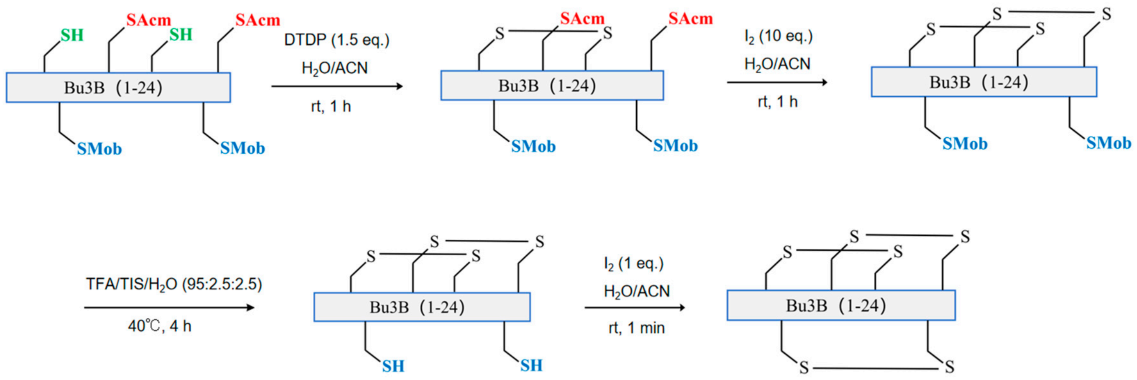

Figure S8. Synthetic scheme of conotoxin BuIIIB.

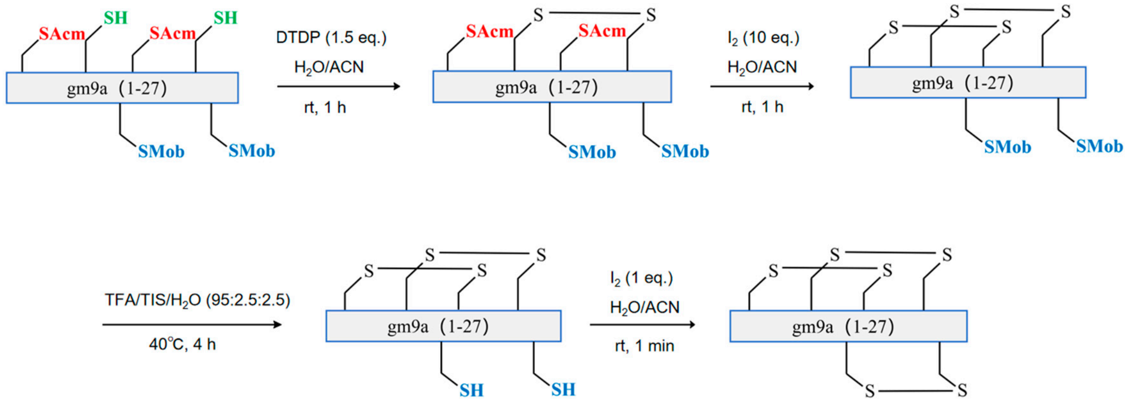

Figure S9. Synthetic scheme of conotoxin gm9a.

3. HPLC chromatograms and CD Spectra

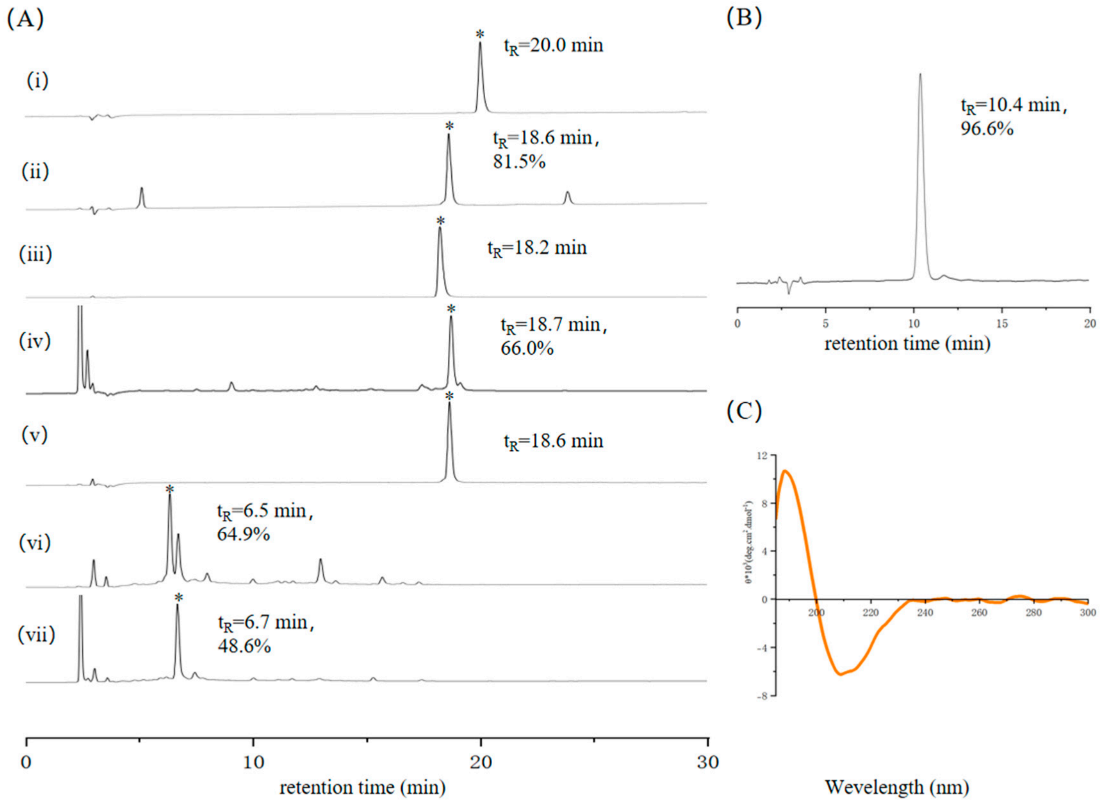

Figure S10. Synthesis of conotoxin KIIIA. (**A**) HPLC chromatograms of the reaction progression. (i) Linear peptide with one pair of free thiols and two pairs protected by S-Mob and S-Acm groups; (ii) added DTDP to facilitate the formation of the first disulfide bond; (iii) monodisulfide intermediate; (iv) the second disulfide bond was formed through I_2-mediated S-Acm oxidation; (v) intermediate with two disulfide bonds; (vi) treated with TFA/TIS/H_2O (95:2.5:2.5) at 45 ℃ for 18 h to remove the S-Mob groups; (vii) added a small amount of iodine to the reaction products in (vi). (**B**) The HPLC chromatogram of synthetic KIIIA. (**C**) CD spectrum of the synthetic KIIIA.

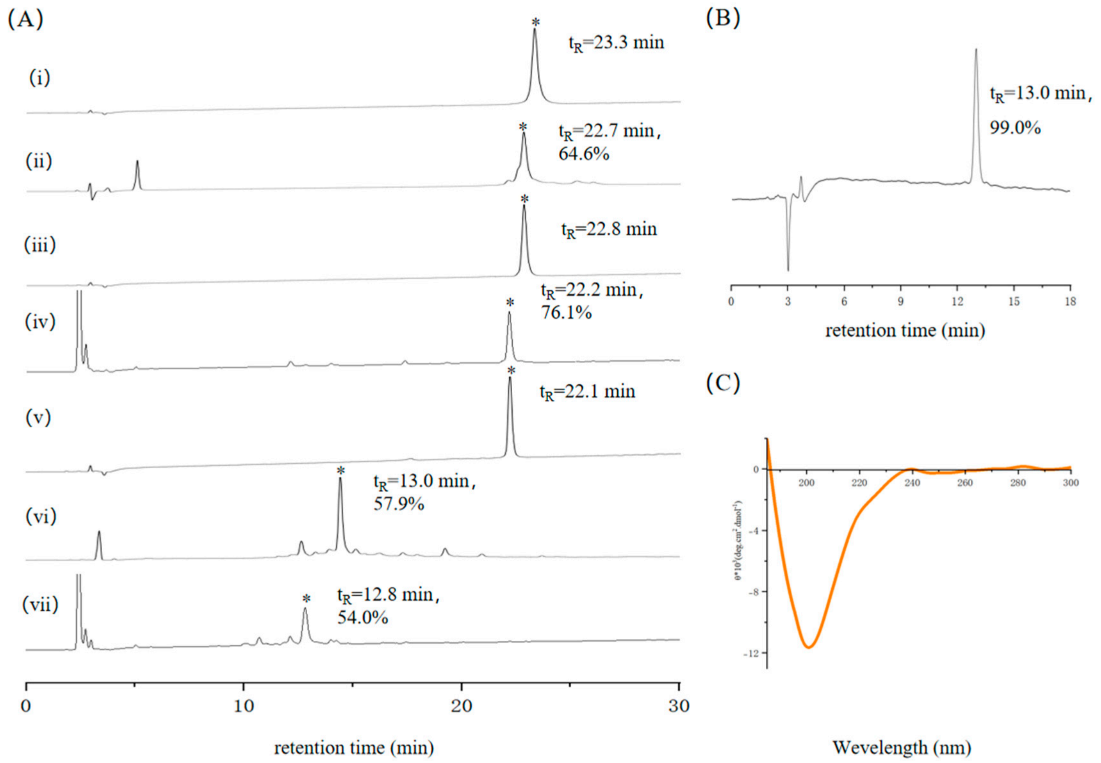

Figure S11. Synthesis of conotoxin gm9a. (**A**) HPLC chromatograms of the reaction progression. (i) Linear peptide with one pair of free thiols and two pairs protected by S-Mob and S-Acm groups; (ii) added DTDP to facilitate the formation of the first disulfide bond; (iii) monodisulfide intermediate; (iv) the second disulfide bond was formed through I_2-mediated S-Acm oxidation; (v) intermediate with two disulfide bonds; (vi) treated with TFA/TIS/H_2O (95:2.5:2.5) at 40 ℃ for 4 h to remove the S-Mob groups; (vii) added a small amount of iodine to the reaction products in (vi). (**B**) The HPLC chromatogram of synthetic gm9a. (**C**) CD spectrum of the synthetic gm9a.

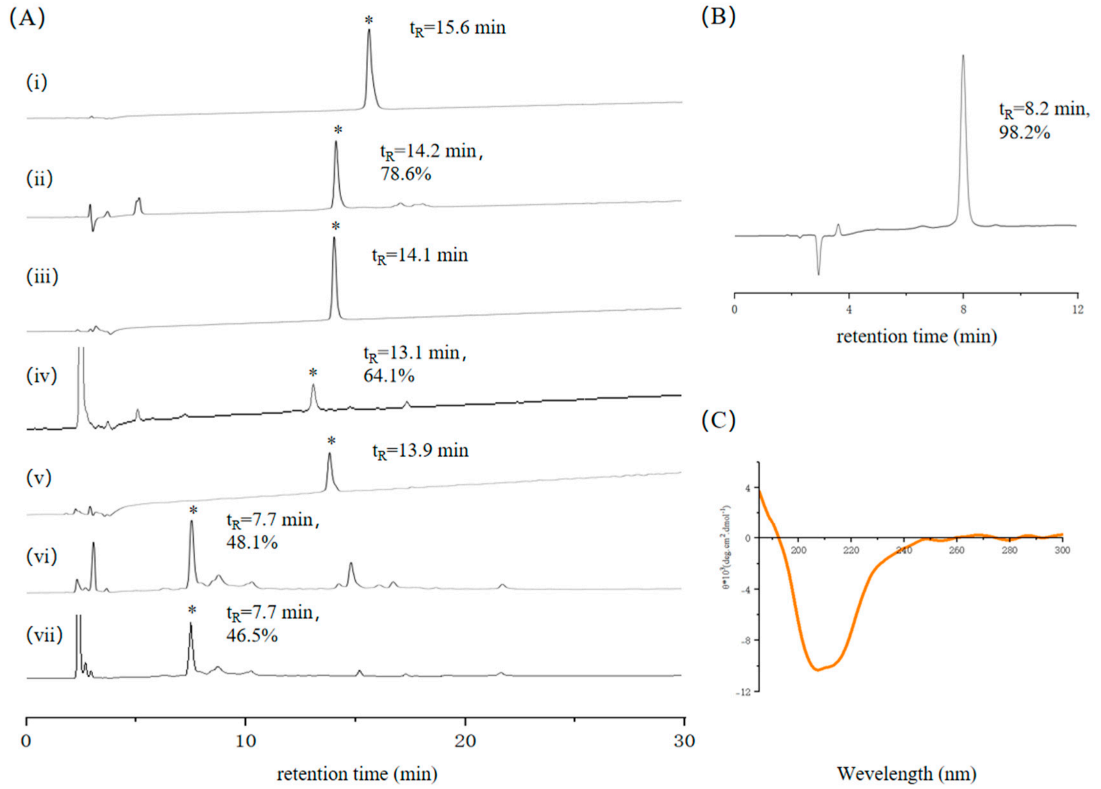

Figure S12. Synthesis of conotoxin BuIIIB. (**A**) HPLC chromatograms of reaction progression. (i) Linear peptide with one pair of free thiols and two pairs protected by S-Mob and S-Acm groups; (ii) added DTDP to facilitate the formation of the first disulfide bond; (iii) monodisulfide intermediate; (iv) the second disulfide bond was formed through I_2-mediated S-Acm oxidation; (v) intermediate with two disulfide bonds; (vi) treated with TFA/TIS/H_2O (95:2.5:2.5) at 40 °C for 4 h to remove the S-Mob groups; (vii) added a small amount of iodine to the reaction products in (vi). (**B**) The HPLC chromatogram of synthetic BuIIIB. (**C**) CD spectrum of the synthetic BuIIIB.

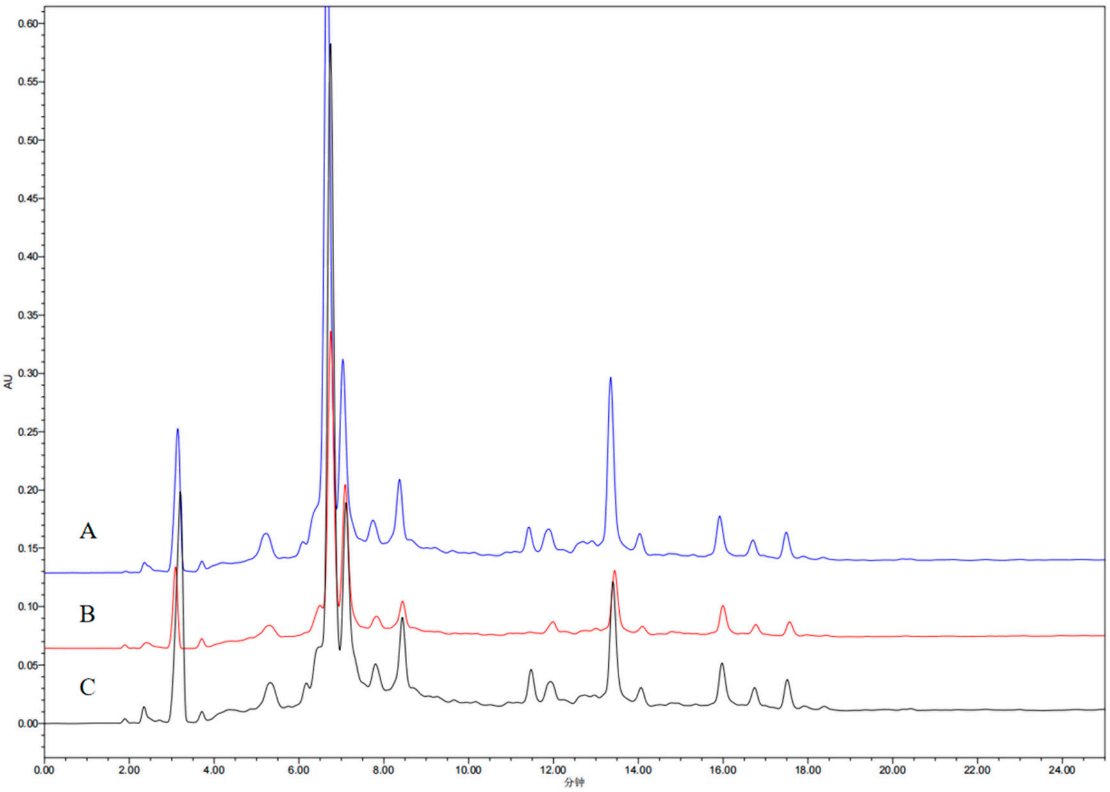

Figure S13. HPLC analysis of KIIIA reactions for S-Mob removal at different temperatures. A. After incubation for 24 h at 40 °C; B. after incubation for 18 h at 45 °C; C. after incubation for 12 h at 50 °C.

4. HPLC of Crude Peptides

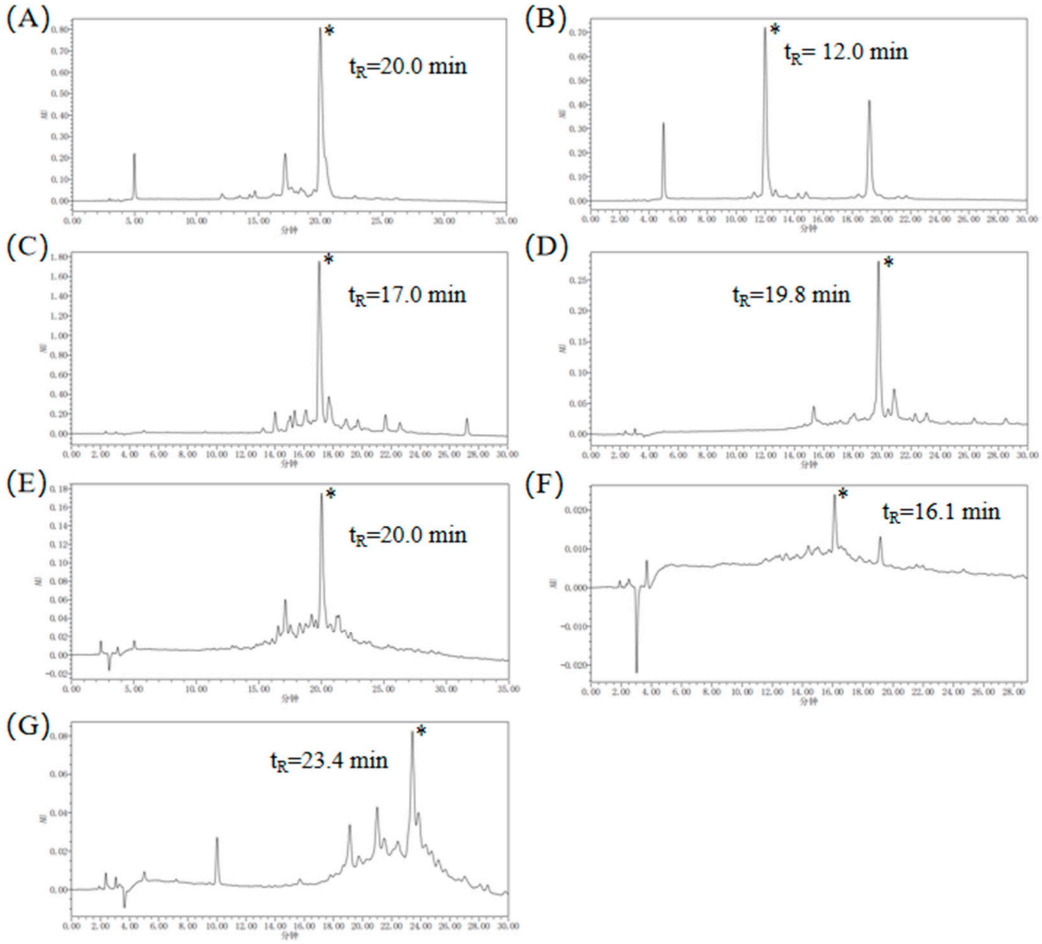

Figure S14. HPLC traces of crude peptides after treatment with reagent K. (**A**) Cz1107-Mob, 55.2% yield. (**B**) Cz1107-Acm, 49.4% yield. (**C**) reg3b, 35.0% yield. (**D**) KIIIA, 38.0% yield. (**E**) MVIIA, 26.8% yield. (**F**) BuIIIB, 22.6% yield. (**G**) gm9a, 20.0% yield.

5. 1H NMR of MVIIA and reg3b

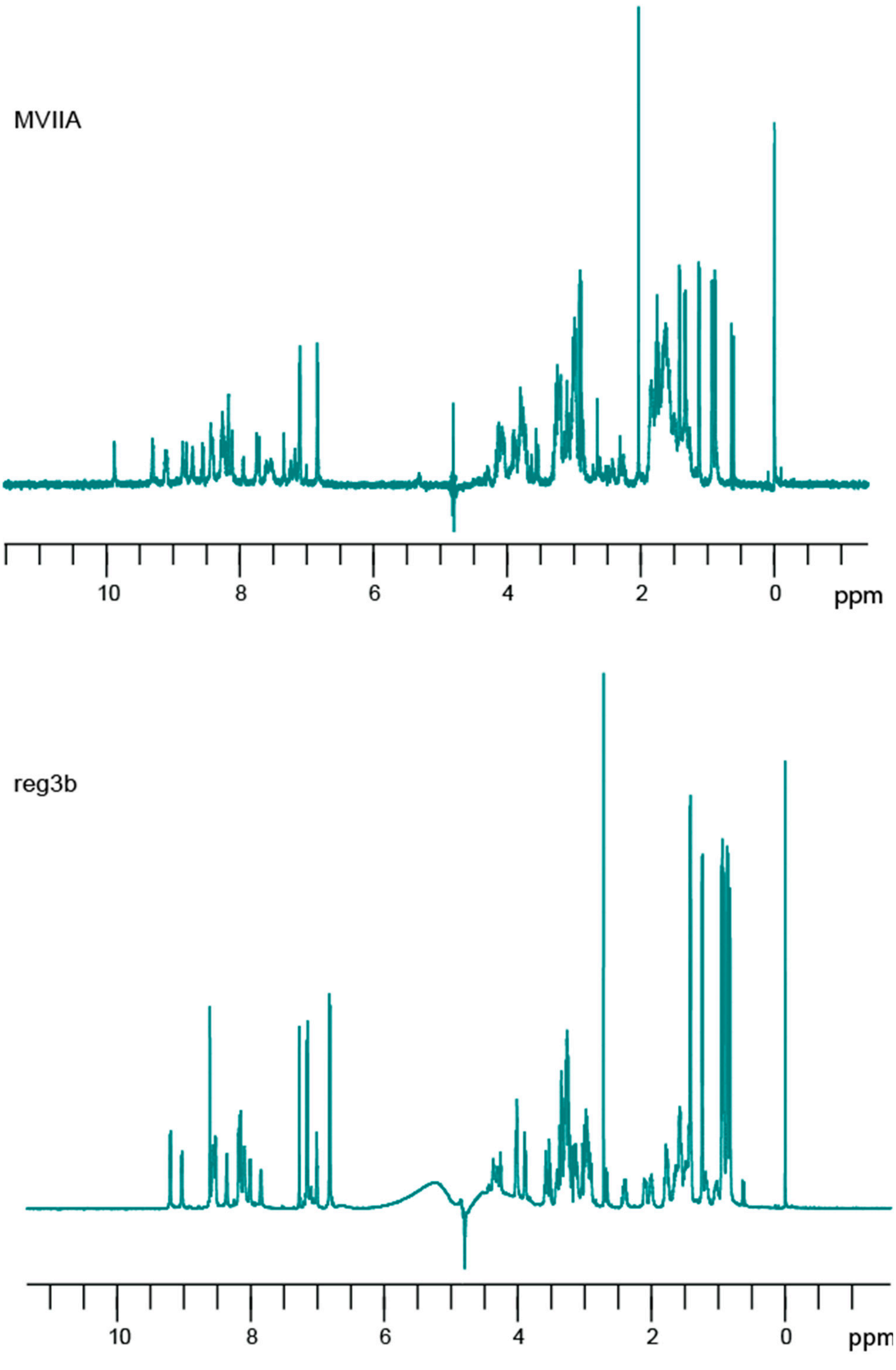

Figure S15. 1H NMR of MVIIA and reg3b.

6. References

23. Franco, A.; Dovell, S.; Möller, C.; Grandal, M.; Clark, E.; Marí, F. Structural plasticity of mini-M conotoxins–expression of all mini-M subtypes by Conus regius. The FEBS journal. **2018**, 285(5), 887-902.

31 Mohan, M.K.; Abraham, N.; Jayaseelan, B.F.; Ragnarsson, L.; Lewis, R.J.; Sarma, S.P. Structure and allosteric activity of a single-disulfide conopeptide from Conus zonatus at human $\alpha3\beta4$ and $\alpha7$ nicotinic acetylcholine receptors. Journal of Biological Chemistry. **2020**, 295(20), 7096-7112.

32 Lin, J.; Chen, S.; Butt, U.D.; Yan, M.; Wu, B. A comprehensive review on ziconotide. **2024**.

33 Zhao, Z.; Pan, T.; Chen, S.; Harvey, P.J.; Zhang, J.; Li, X.; Yu, R. Design, synthesis, and mechanism of action of novel μ-conotoxin KIIIA analogues for inhibition of the voltage-gated sodium channel Nav1.7. Journal of Biological Chemistry. **2023**, 299(4).

34 Holford, M.; Zhang, M.M.; Gowd, K.H.; Azam, L.; Green, B.R.; Watkins, M.; Olivera, B.M. Pruning nature: Biodiversity-derived discovery of novel sodium channel blocking conotoxins from Conus bullatus. Toxicon. **2009**, 53(1), 90-98.

35 Miles, L.A.; Dy, C.Y.; Nielsen, J.; Barnham, K.J.; Hinds, M.G.; Olivera, B.M.; Norton, R.S. Structure of a novel P-superfamily spasmodic conotoxin reveals an inhibitory cystine knot motif. Journal of Biological Chemistry. **2002**, 277(45), 43033-43040.
